# Electric field-driven building blocks for introducing multiple gradients to hydrogels

**DOI:** 10.1007/s13238-020-00692-z

**Published:** 2020-02-12

**Authors:** Gang Xu, Zhaozhao Ding, Qiang Lu, Xiaoyi Zhang, Xiaozhong Zhou, Liying Xiao, Guozhong Lu, David L Kaplan

**Affiliations:** 1 Department of Orthopedics, The Second Affiliated Hospital of Soochow University, Suzhou, 215000 China; 2 Department of Burns and Plastic Surgery, Engineering Research Center of the Ministry of Education for Wound Repair Technology, The Affiliated Hospital of Jiangnan University, Wuxi, 214041 China; 3 National Engineering Laboratory for Modern Silk & Collaborative Innovation Center of Suzhou Nano Science and Technology, Soochow University, Suzhou, 215123 China; 4 Department of Orthopedics, Affiliated Hospital of Xuzhou Medical University, Lianyungang, 222061 China; 5 Department of Biomedical Engineering, Tufts University, Medford, MA 02155 USA

**Keywords:** silk, building blocks, gradients, hydrogel, tissue regeneration

## Abstract

Gradient biomaterials are considered as preferable matrices for tissue engineering due to better simulation of native tissues. The introduction of gradient cues usually needs special equipment and complex process but is only effective to limited biomaterials. Incorporation of multiple gradients in the hydrogels remains challenges. Here, beta-sheet rich silk nanofibers (BSNF) were used as building blocks to introduce multiple gradients into different hydrogel systems through the joint action of crosslinking and electric field. The blocks migrated to the anode along the electric field and gradually stagnated due to the solution-hydrogel transition of the systems, finally achieving gradient distribution of the blocks in the formed hydrogels. The gradient distribution of the blocks could be tuned easily through changing different factors such as solution viscosity, which resulted in highly tunable gradient of mechanical cues. The blocks were also aligned under the electric field, endowing orientation gradient simultaneously. Different cargos could be loaded on the blocks and form gradient cues through the same crosslinking-electric field strategy. The building blocks could be introduced to various hydrogels such as Gelatin and NIPAM, indicating the universality. Complex niches with multiple gradient cues could be achieved through the strategy. Silk-based hydrogels with suitable mechanical gradients were fabricated to control the osteogenesis and chondrogenesis. Chondrogenic-osteogenic gradient transition was obtained, which stimulated the ectopic osteochondral tissue regeneration *in vivo*. The versatility and highly controllability of the strategy as well as multifunction of the building blocks reveal the applicability in complex tissue engineering and various interfacial tissues.

**Electronic supplementary material:**

The online version of this article (10.1007/s13238-020-00692-z) contains supplementary material, which is available to authorized users.

## Introduction

Native tissues such as skin, bone, nerve and muscle have multiple gradients to regulate cell behaviors and guide tissue functions (Lu and Thomopoulos, 2013; Vedadghavami et al., 2017; Di Donato et al., 2018; Li et al., 2018; Radhakrishnan et al., 2018; Wu et al., 2018). Both biochemical and physical cues including compositions, growth factors, stiffness and topography gradients play critical rules in controlling cell fate and tissue function (Oh et al., 2016; Naskar et al., 2017; Hubka et al., 2019). Strategies were developed to endow the engineered tissues with these different gradients *in vitro* for achieving functional recovery of damaged tissues (Han et al., 2016; Pogoda et al., 2017; Kokkinis et al., 2018). For instance, Microfluidic devices, 3D printing and magnetic field are used to fabricate gradients in biomaterials (Bracaglia et al., 2017; Moller et al., 2017; Zhang et al., 2017; Kokkinis et al., 2018; Li et al., 2018). These approaches usually need special apparatus and rigorous parameters and are only feasible for specific materials. Photopatterning is also used widely to introduce both physical and chemical gradients in which photoresponsivity is a prerequisite for the treated systems (Gao et al., 2019). Recently, buoyancy-driven gradients of various cargos were achieved for different biomaterials, suggesting a more versatile strategy of fabricating bioactive biomaterials used in complex tissue regenerations (Li et al., 2019). However, finer regulations of these gradients are still required to optimize the functional recovery of tissues. Although plenty of approaches have been developed to form biomaterial gradients (Wang et al., 2018a; Yang et al., 2018; Yin et al., 2018; Gao et al., 2019; Li et al., 2019), little studies could introduce the gradients of multiple cargos simultaneously due to the interference of different cargos in fabrication processes. A gap remains for the biomaterials with gradients and native microenvironments of tissues *in vivo*.

Silk fibroin (SF) is a versatile natural biomaterial as matrices for tissues including skin, nerve, blood vessel, cartilage and bone (Ding et al., 2016b; Lu et al., 2018; Wang et al., 2018b), and also as carriers to load growth factors, macromolecules and small molecule drugs (Shen et al., 2016; Aigner et al., 2018). Recently, beta-sheet rich SF nanofibers (BSNF) were assembled in aqueous solutions in our group, exhibiting superior biocompatibility and loading drug capacity to previous SF materials (Lu et al., 2011; Wu et al., 2016). BSNF as reinforcing nanofibers could be introduced to different hydrogels and scaffolds to tune the mechanical cues finely, resulting in controllable differentiation behaviors of stem cells (Liu et al., 2019; Lu et al., 2019). Different cargos such as small molecules, growth factors, graphene sheets and gold nanoparticles were loaded on the nanofibers, which provided tunable physical and biochemical cues for cells and tissues (Ding et al., 2016b; Wu et al., 2016; Zhang et al., 2018; Xu et al., 2019). The BSNF could move directly in electric field and form aligned hydrogels (Lu et al., 2016; Lu et al., 2018). Therefore, BSNF is power building blocks to introduce multiple cargos to hydrogels. The movement of BSNF in the electric field was influenced by electric intensity and solution viscosity (Lu et al., 2018; Wang et al., 2018b). It is possible to form gradients through tuning BSNF movement in electric field and then solidifying the gradients after hydrogel formation.

Here, electric field-driven silk nanofiber building blocks were applied to introduce gradients into hydrogels. The movement rate of the building blocks was determined by both of electric field intensity and the changed viscosity in solution-hydrogel transition process, resulting in gradient distribution in the formed hydrogels. High tunability was achieved through changing the electric intensity and crosslinking parameters simply. As an effective reinforcement nanofiber, the BSNF distributed with gradient in different hydrogel systems, which provided continuous mechanical gradient signal. Aligned gradients were also given simultaneously since the nanofiber blocks oriented under electric field. Different functional cargos such as drugs could be loaded on the blocks, further enriching multiple cue gradients inside the same hydrogel systems. To the best of our knowledge, it is the first time to provide tunable gradients of multiple cargos simultaneously in hydrogels. The simple process, high tunability of the system, strong capacity of loading gradients of multiple cargos and university for various hydrogels suggest the strategy as a generalized approach for introducing gradients effectively to tissue engineering.

## Results and discussion

**Figure 1:**
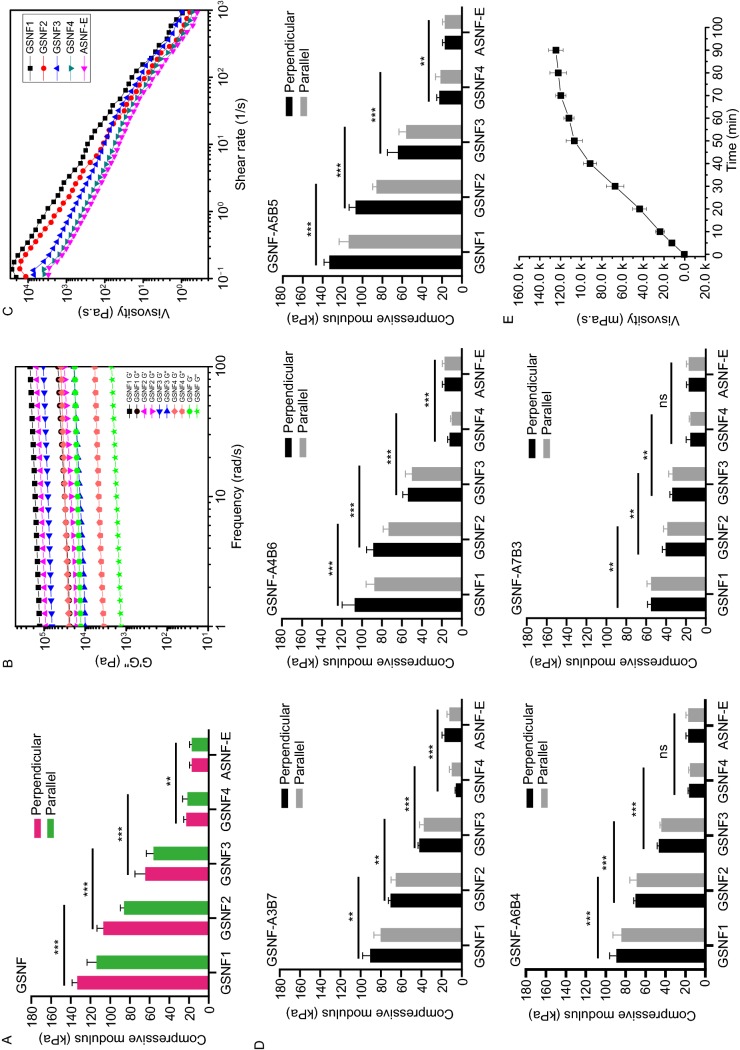

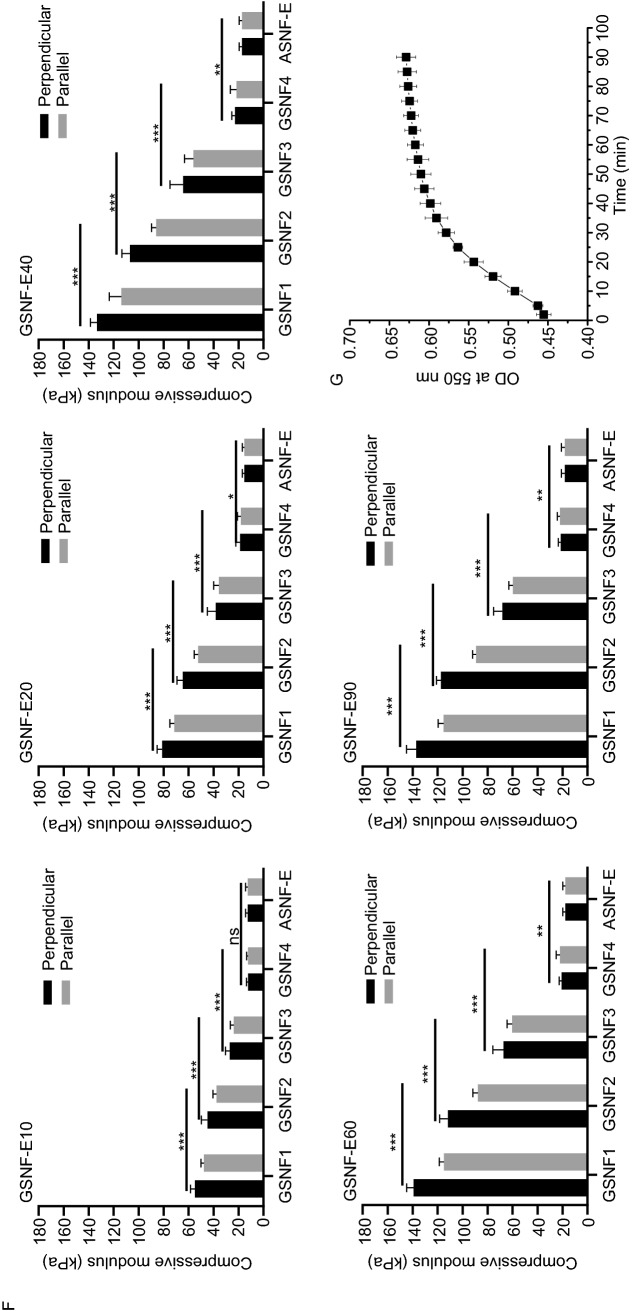
**Characterization of the hydrogels with mechanical gradient cues.** (A) anisotropic and gradient mechanical properties of the GSNF hydrogels; (B) Gradient visco-elasticity of the GSNF hydrogels; (C) Viscosity properties of the GSNF hydrogels; (D) Tunable mechanical gradients of the GSNF hydrogels through tuning the ratio of ASNF and BSNF; (E) Viscosity of the composite solutions (ASNF and BSNF) with HRP crosslinking from 0 min to 90 min; (F) Tunable mechanical gradients of the GSNF hydrogels through tuning the HRP crosslinking time of ASNF; (G) OD values of the composite hydrogels (ASNF and BSNF) with HRP crosslinking from 0 min to 90 min at 550 nm. The samples were as follows: ASNF, the amorphous silk fibroin nanofiber hydrogel; BSNF, the beta-sheet rich silk nanofiber hydrogel; GSNF, ASNF and BSNF composite hydrogels with gradients; GSNF1, GSNF2, GSNF3 and GSNF4, the four parts of the GSNF hydrogels equally divided along the electrical field direction from the anode to the cathode; ASNF-E, the HRP-crosslinked ASNF hydrogel; GSNF-E10, GSNF-E20, GSNF-E40, GSNF-E60 and GSNF-E90, the composite hydrogels that the HRP-crosslinking time before electrical field treatment was 10 min, 20 min, 40 min, 60 min and 90 min, respectively. GSNF-A3B7, GSNF-A4B6, GSNF-A5B5, GSNF-A6B4 and GSNF-A7B3, the volume ratios of ASNF and BSNF in the GSNF hydrogels were 3:7, 4:6, 5:5, 6:4, and 7:3, respectively

As a proof of concept, beta-sheet rich silk nanofiber solutions (BSNF) were blended with amorphous silk nanofiber solutions (ASNF) where BSNF was used as reinforcement fibers to provide mechanical and oriented gradients while ASNF was crosslinked to form hydrogel matrices. Without the crosslinking of ASNF, the BSNF moved gradually to the anodes and formed hydrogels after 30 min under the electric fields with voltage of 50 V. If ASNF was crosslinked simultaneously when the blended solutions were treated under electric field, the migration rate of BSNF was slowed down due to the increase of viscosity and finally stagnated following the solidification of ASNF (Fig. S1). The joint action of crosslinking and electric field resulted in the gradient distribution of BSNF in the ASNF hydrogels, which further endowed the hydrogels with gradient mechanical properties. BSNF was also aligned under electric field, providing orientation gradient simultaneously. As a typical sample, 2% of ASNF and 2% of BSNF solutions were blended at volume ratio of 1:1 to form the mixed solution with silk concentration of 2%. The crosslinking of ASNF was trigged after H_2_O_2_ introduction and further treated under the electrical field (50 V). After 15 min, the crosslinked hydrogels could be cured fully where the amount of BSNF gradually decreased from the anode to the cathode. To study the gradients, the hydrogels were cut to four parts along the electrical field direction and termed GSNF1, GSNF2, GSNF3 and GSNF4 from the anode to the cathode. As a control, ASNF hydrogel (2 wt%) was also crosslinked under same conditions without the introduction of BSNF. When the hydrogels were compressed in a direction perpendicular to the electric field, gradually increased mechanical stiffness from 22.9 kPa to 133.4 kPa was achieved for different areas of the hydrogels from the cathode to the anode, suggesting successive mechanical gradients (Fig. 1A). Similar to our previous electric field-treated BSNF hydrogels, the composite hydrogels exhibited mechanical anisotropy (Lu et al., 2016). For the formed hydrogel near the anode, the modulus decreased from 133.4 kPa to 113.9 kPa when the samples were compressed in a direction parallel to the electric field (Table S1). The degree of mechanical anisotropy also showed gradient changes. The ratio of the two moduli (perpendicular to and parallel to the electric field respectively) gradually decreased from 1.17 to 1 for the areas near the anode and near the cathode (Table S2), which should result from the gradient distribution of aligned BSNF in the hydrogels.

Since the movement of BSNF could be easily controlled by solution viscosity and electric field voltage in our systems, the gradients of orientation and mechanical cue showed tunability. For example, the beginning viscosity of the mixed solutions under electric field treatment could be regulated through changing the ratio of ASNF and BSNF. When the ratios of ASNF and BSNF were 3:7, 4:6, 6:4, and 7:3, respectively, the moduli gradually increased from 6.0 kPa to 90.8 kPa, from 12.4 kPa to 107.3 kPa, from 16.4 kPa to 89.4 kPa and from 15.7 kPa to 55.3 kPa when the mechanical properties of the hydrogels were measured from the anode to the cathode under the direction perpendicular to the electric field (Fig. 1D). The ratio of the two moduli (perpendicular to and parallel to the electric field respectively) also exhibited corresponding gradient changes for the different hydrogels. When the ratios of ASNF and BSNF were 3:7, 4:6, 6:4, and 7:3 in the formed hydrogels, the ratios of the two moduli correspondingly changed from 1.13, 1.22, 1.06 and 1.03 to 1 for the hydrogel areas near the anode and near the cathode, respectively (Table S2). The gradients of mechanical cue and orientation could be tuned through tuning the trigging times of crosslinking and electrical field treatment. Before the mixed solution was electrified, the ASNF could be crosslinked for different times to change the viscosity of the solution. When the pre-crosslinking time was 10 min, 20 min, 40 min, and 60 min, respectively, the viscosity of the solutions before electric field treatment was changed to 23.9 Pa.s, 43.7 Pa.s, 91.5 Pa.s and 112 Pa.s (Fig. 1E), correspondingly. After the electric field treatment, the moduli gradually increased from 12.4 kPa to 54.9 kPa, from 18.8 kPa to 81.2 kPa, from 20.7 kPa to 139.3 kPa and from 21.5 kPa to 137.1 kPa when the mechanical properties of the hydrogels were measured from the anode to the cathode under the direction perpendicular to the electric field (Fig. 1F). All these results suggested that both mechanical and oriented gradients could be regulated through tuning multiple factors simply, superior to gradient hydrogel systems reported previously (Wang et al., 2018b; Yang et al., 2018; Gao et al., 2019). The mechanical gradients of the hydrogels could be cover different tissues including skin, muscle, cartilage and bone, suggesting their promising applications across these tissues (Nonoyama et al., 2016; Oh et al., 2016; Lu et al., 2018; Yin et al., 2018).

**Figure 2:**
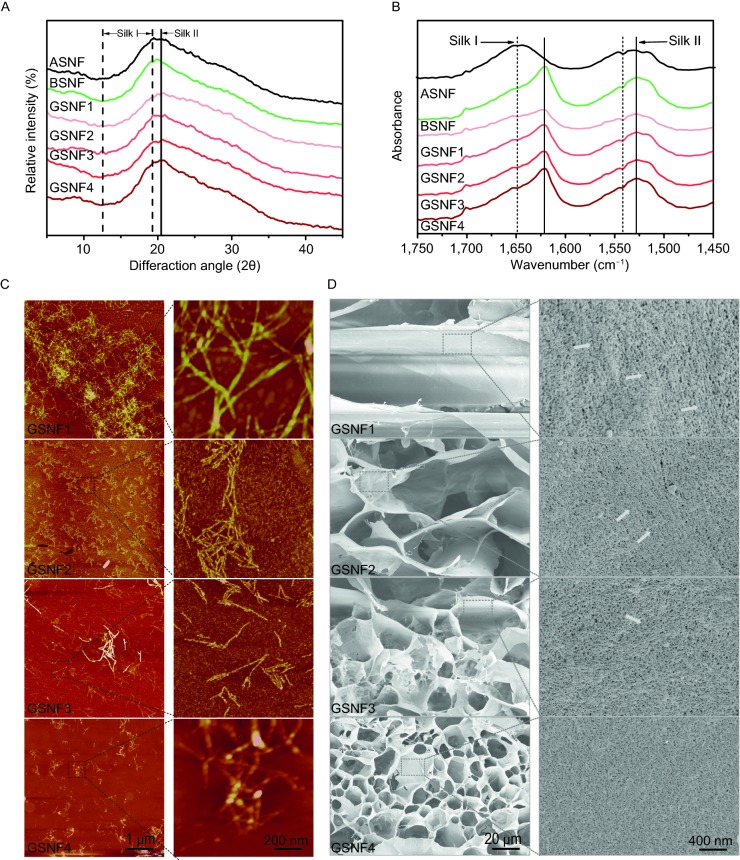
**Secondary structure and microscopic morphology of the silk nanofiber hydrogels with mechanical gradient cues.** (A) XRD and (B) FTIR spectra of different areas of the hydrogels with various mechanical properties; (C) AFM images of different area of the hydrogels with various mechanical cues. Higher amount of BSNF appeared at the sites with stiffer mechanical properties; (D) SEM images of different area of the hydrogels with various mechanical cues. The images exhibited better anisotropic morphologies at sites with stiffer mechanical properties. White arrows indicated the aligned structures

A key factor that determines the gradient in our hydrogel systems is the distribution of BSNF in the hydrogel matrices. Contrary to ASNF that could be crosslinked effectively with HRP but remain inactive under electric field, BSNF that remained inert to HRP crosslinking could migrated to the anode directionally, resulting in its gradient distribution. ASNF with length of 100–400 nm was mainly composed of amorphous states while the beta-sheet content in BSNF (length of about 1–2 μm) was above 50%. Both XRD and FTIR indicated gradual increase of beta-sheet content from the area near the cathode to that near the anode for the formed hydrogels, suggesting gradient higher content of BSNF near the anode (Fig. 2A and 2B). Fourier self-deconvolution of the amide I region confirmed the gradient distribution of BSNF. The beta-sheet content in pure ASNF was 25.29% and increased to 55.47% for pure BSNF (Table S3). When ASNF and BSNF were blended to form homogeneous solutions at volume ratio of 1:1, beta-sheet content of the freeze-dried blend solution was 40.5%. After the solution was crosslinked to form hydrogel under electric field, the hydrogels collected from different areas from the cathode to the anode exhibited the increase of beta-sheet structure from 29.57% to 51.55%. AFM images of different areas further revealed more BSNF appeared near the anode while fewer BSNF could be found from the hydrogel near the cathode (Fig. 2C). SEM images indicated the gradual decline of hierarchy and orientation from GSNF1 to GSMF4, and the gradual increase of network structure (Fig. 2D). Similar to pure electric field-treated BSNF hydrogels (Ding et al., 2017.09), BSNF nanofibers in the composite hydrogels had preferable orientation parallel to the electric field. Higher contents of BSNF near the anode area facilitated the formation of more aligned layers, which resulted in the gradient of aligned structures and also the changes of anisotropic mechanical properties. All these results confirmed the critical function of BSNF distribution in regulated the gradients of the hydrogels.

**Figure 3:**
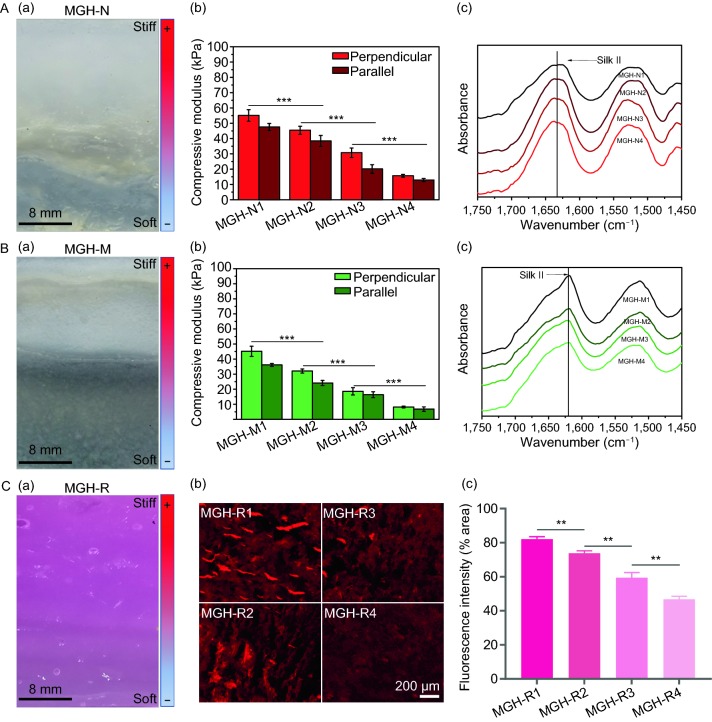
**Introduction of gradient cues into various hydrogel systems based on the BSNF blocks**. (A) NIPAM-based hydrogels with mechanical gradient cues: (a) macrographic images of NIPAM hydrogels with mechanical gradient cues, (b) The gradient and anisotropic mechanical properties of the hydrogels, (c) FTIR spectral of the hydrogels at different areas; (B) Gel-MA-based hydrogels with mechanical gradient cues: (a) macrographic images of Gel-MA hydrogels with mechanical gradient cues, (b) The gradient and anisotropic mechanical properties of the hydrogels, (c) FTIR spectral of the hydrogels at different areas; (C) Silk nanofiber hydrogels with rhodamine gradients: (a) macrographic images of silk hydrogels with rhodamine gradients, (b) Confocal microscopy images of different areas of rhodamine-loaded hydrogels, (c) Rhodamine fluorescence intensity of different areas of rhodamine-loaded hydrogels. The samples were as follows: MGH-N, NIPAM-BSNF composite hydrogels with gradient cues; MGH-M, GEL-MA-BSNF composite hydrogels with gradient cues; MGH-R, rhodamine-loaded ASNF-BSNF composite hydrogels with gradient cues

Unlike previous gradient fabrication methods that are usually effective for certain materials or cargos (Ding et al., 2017.09; Ko et al., 2018), BSNF, as a universal reinforcement nanofiber, could be used to tune the mechanical properties of different biomaterial systems. For example, the composite solutions of BSNF and NIPAM could be cross-linked by APS and formed hydrogels with mechanical and oriented gradients under electric fields (Fig. 3A). BSNF were also added to Gel-MA hydrogel systems and triggered the photo-crosslinking under electric field (Fig. 3B). Similarly, the composite hydrogels with mechanical and oriented gradients were prepared, confirming the versatility of the strategy. Previous studies revealed that BSNF was a superior carrier for both hydrophilic and hydrophobic cargos as well as macromolecule/small molecule drugs (Wu et al., 2016; Hassani Besheli et al., 2017; Ding et al., 2017.09). To determine whether more gradients of chemical cargos could be introduced to the hydrogels, rhodamine as a model drug, was loaded on the BSNF nanofibers and blended with ASNF solutions, and then was crosslinked with HRP under the electric field through our established ASNF-BSNF systems (Fig. 3C). Gradient distribution of rhodamine was visualized in the formed hydrogel, which revealed the feasibility of introducing chemical gradients simultaneously. Therefore, the versatile functions of BSNF made it feasible to create multiple gradients with single block, which is impossible for other reported systems (Bracaglia et al., 2017; Wang et al., 2018b; Li et al., 2019). It is important to note that our strategy also shows superior tunability, providing versatile options to engineer complex niches according to the requirements of different tissues.

To demonstrate the advantages of electric field-driven blocks in tissue engineering, the composite nanofiber hydrogels with suitable mechanical gradients were fabricated to facilitate the engineering of the osteochondral interface. Challenges remain to regenerate osteochondral interface due to the complex gradient niches that was difficult to be imitated for most hydrogel systems (Levingstone et al., 2016; Liao et al., 2017; Di Donato et al., 2018; Yang et al., 2018). Cell fates could be determined by mechanical cues (Han et al., 2016; Wang et al., 2018a; Liu et al., 2019). The cells sense mechanical signals and secret different growth factors to control the differentiation, proliferation and migration behaviors (Berger et al., 2017; Pogoda et al., 2017). It is considered that the modulus of 18–28 kPa could induce the differentiation of stem cells to chondrocytes while the modulus of above 28 kPa would stimulate the osteo-differentiation (Engler et al., 2006; Ding et al., 2017.09). Therefore, the hydrogels with successive stiffness gradients from 20 kPa to 130 kPa were fabricated through the ASNF-BSNF composite hydrogel systems. Besides the mechanical gradient, better aligned structures formed in the areas with higher stiffness, which would influence the cell behavior *in vitro* and *in vivo*.

**Figure 4:**
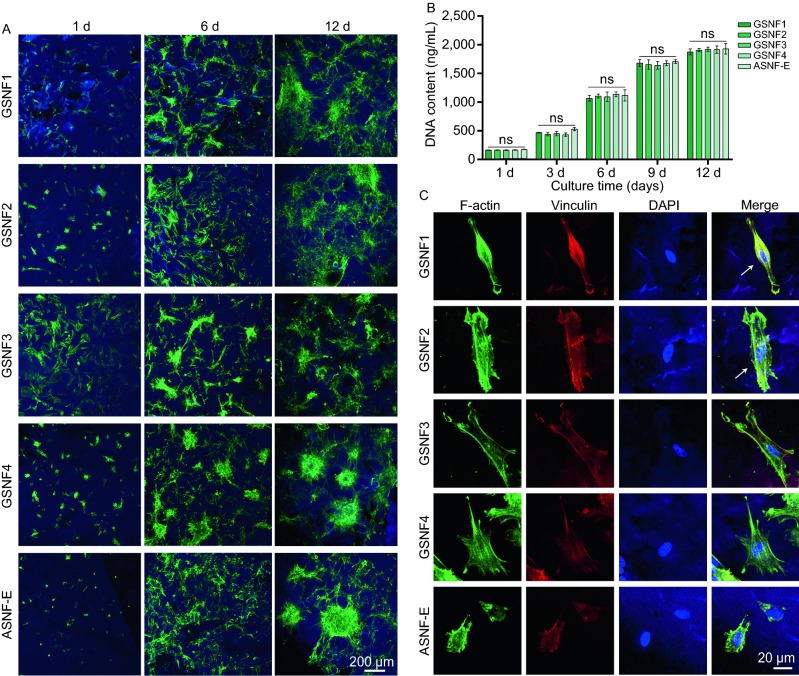
**Cytocompatibility and cell adhesion on the hydrogels with mechanical cues**. (A) Confocal microscopy images of BMSCs on the hydrogels when cultured for 1, 6, and 12 days; (B) BMSC proliferation on different hydrogels when cultured for 1, 3, 6, 9 and 12 days; (C) immunofluorescence staining of adhered BMSCs on the hydrogels for 24 h. Nuclei is stained in blue. Vinculin is stained in red, and F-actin is stained in green. The white arrows indicate the oriented spreading of cells. ns means none statistically significant

The gradient hydrogels were divided into four transverse sections along the direction from the anode to the cathode, and termed GSNF1, GSNF2, GSNF3 and GSNF4, respectively. The HRP-crosslinked hydrogels of ASNF solutions were fabricated and used as a control. As shown in Table S1, the moduli of these five hydrogels were 17 kPa, 23 kPa, 64 kPa, 107 kPa, and 133 kPa, respectively. Both ASNF and BSNF were composed of nanofibers and had good cytocompatibility (Fig. 4A). All the BSMCs cultured on different hydrogel samples exhibited similar proliferation behaviors, suggesting their similar cytocompatibility without significant difference (Fig. 4B). The results confirmed that ASNF and BSNF composite hydrogels were good matrices for tissue engineering. As expected, the oriented gradients in the hydrogels could influence the adhesion and aggregation behaviors. When the cells cultured on different hydrogels with gradually better aligned structures for 1 d, the morphology of the adhered cells changed from polygonal shape to elongated spindle structure (Fig. 4C). After cultured on the hydrogels for 12 days, the cells aggregated to form spheres on the hydrogels with inferior aligned structures but showed the oriented cell network on the hydrogels with better aligned structures (Fig. 4A).

**Figure 5:**
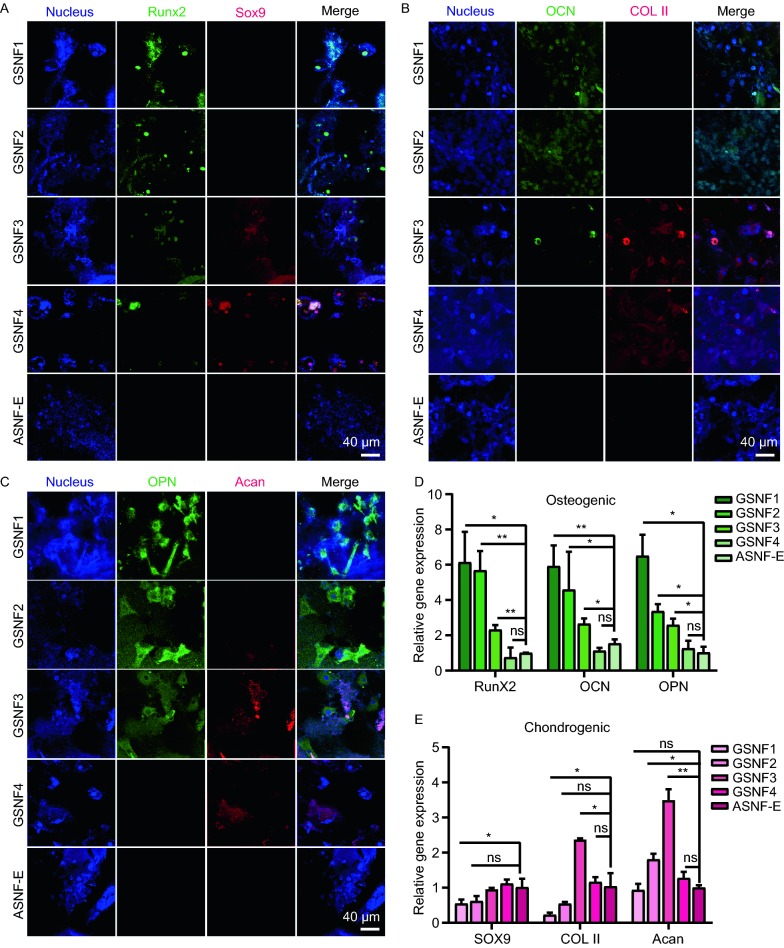
**Osteogenic and chondrogenic differentiation of BMSCs when cultured on the hydrogels with mechanical gradient cues for 28 days**. (A–C) Immunofluorescence staining for Runx2/SOX9, OCN/COL II, and OPN/Acan of BMSCs. Nuclei are stained in blue, Runx2, OCN, and OPN are stained in green, SOX9, COL II and Acan are stained in red. scale bar 40 μm; (D and E) mRNA levels of Runx2/SOX9, OCN/COL II, and OPN/Acan of BMSCs on the hydrogels. The expression of KDR is normalized to GAPDH. *P ≤ 0.05, **P ≤ 0.01, ***P ≤ 0.001 and ns means none statistically significant

Then BMSCs were cultured on the hydrogels for 28 days to evaluate differentiation behaviors. Immunofluorescence staining for different key proteins related to osteogenesis and chondrogenesis including Runx2, OPN, OCN, COL II, Acan, and SOX9 was used to reveal gradient chondrogenic and osteogenic differentiation behaviors of BMSCs on the hydrogels (Fig. 5A–C). When the cells were cultured on the control hydrogels (stiffness 17 kPa), no significant expression of osteogenic and chondrogenic markers appeared, suggesting that the hydrogels had no osteogenic and chondrogenic capacity. Gradient increased stiffness of the hydrogels (GSNF1, GSNF2, GSNF3 and GSNF4) resulted in different osteogenic and chondrogenic tendency of the cells, which revealed effective function of mechanical gradient cues. Gradually higher expression of Runx2, OPN, OCN was achieved following the increase of hydrogel stiffness, and then optimized for the hydrogel with highest modulus of 133 kPa while the expression of chondrogenic markers (COL II, Acan, and SOX9) for the cells increased when the stiffness of the hydrogels increased from 23 kPa to 64 kPa, peaked on the hydrogels with stiffness of 64 kPa and then decreased if the stiffness was further increased to 107 kPa and 133 kPa. The PCR results of the above expression markers further confirmed the different trends of osteogenesis and chondrogenesis. The highest expression of osteogenic markers (Runx2, OCN, and OPN) appeared on the hydrogels with highest modulus (Fig. 5D) while the cells cultured on the hydrogels with stiffness of 64 kPa achieved best expression of chondrogenic markers (SOX9, COL II, and Acan) (Fig. 5E). The results indicated that the anticipated localized osteogenic and chondrogenic responses were achieved through the introduction of mechanical gradients in the hydrogels. The changes of osteogenesis and chondrogenesis were consistent with that happened on the osteochondral interface *in vivo*, superior to most of previous osteochondral tissue engineering systems fabricated through complex processes (Naskar et al., 2017; Zhang et al., 2017; Studle et al., 2018; Yang et al., 2018).

**Figure 6:**
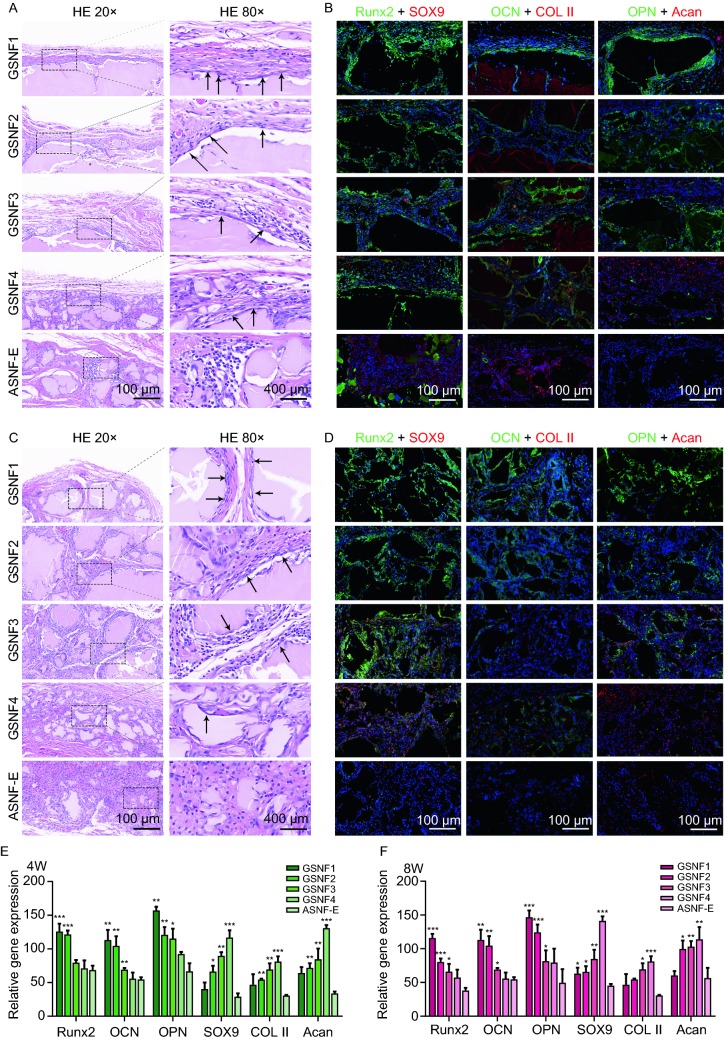
**Heterotopic ossification of hydrogels with gradient mechanical cues*in vivo***. (A) H&E staining of the hydrogels when implanted subcutaneously for 4 weeks, the black arrows indicate the oriented growth of cells around the GSNF hydrogels; (B) Immunofluorescence analysis of osteogenic/chondrogenic-related markers of the hydrogels when implanted subcutaneously for 4 weeks. Cell nuclei are stained in blue, Runx2, OCN, and OPN are stained in green, SOX9, COL II and Acan are stained in red. Scale bar 100 μm; (C) H&E staining of the hydrogels when implanted subcutaneously for 8 weeks; (D) Immunofluorescence analysis of osteogenic/chondrogenic-related markers of the hydrogels when implanted subcutaneously for 8 weeks. Cell nuclei are stained in blue, Runx2, OCN, and OPN are stained in green, SOX9, COL II and Acan are stained in red. Scale bar 100 μm; (E and F) Quantitative analysis of fluorescence intensity of osteogenic/chondrogenic specific proteins when implantation for 4 and 8 weeks. *P ≤ 0.05, **P ≤ 0.01, and ***P ≤ 0.001

To further confirm different osteo-conduction and chondro-conduction of the hydrogels with mechanical gradients, the hydrogels were implanted subcutaneously and evaluate ectopic osteochondral tissue regeneration. After 4 weeks and 8 weeks of implantation, the cells migrated into the hydrogels gradually following the degradation of the hydrogels. Similar to the *in vitro* results, although plenty of cells migrated into the hydrogels, little osteocytes and chondrocytes appeared in the control hydrogels with homogeneous modulus of 17 kPa, suggesting negligible osteochondral induction of the hydrogels (Fig. 6A and 6C). Unlike the control hydrogels, the hydrogels with highest stiffness were mostly occupied by the osteocytes while plenty of chondrocytes were found in the softest hydrogels with modulus of 23 kPa. Then, both chondrocytes and osteocytes existed in the hydrogels with middle stiffness of 64 kPa (Fig. 6B and 6C). The qualitative gene expression results confirmed the gradient chondrogenic-osteogenic transition on the hydrogels with mechanical gradients (Fig. 6E and 6F). Although the optimal chondro-conduction appeared in different areas *in vitro* and *in vivo* (GSNF3 and GSNF4) partly since complex cell-material interaction *in vivo* might be change the stiffness of the hydrogels (Studle et al., 2018), all the results suggested similar distribution of osteocytes and chondrocytes to that native osteochondral tissues *in vivo*. We believed that the mechanical gradients provided suitable cues to tune osteogenic and chondrogenic capacity of the hydrogels. The oriented gradients provided additional benefits to tissue regeneration. The osteocytes aggregated to form aligned structures in the hydrogels with highest stiffness and best oriented structures, which were more similar to native bones (Fig. 6C). Although further work is required to evaluate the function of the hydrogels in osteochondral tissue regeneration through *in situ* defect models, the *in vitro* and *in vivo* gradient osteogenic-chondrogenic capacity as well as effective aligned cues of the hydrogels clearly revealed the superiority of our systems in complex tissue regenerations.

## Conclusions

In summary, multifunctional building blocks, beta-sheet rich silk nanofibers, were introduced to pattern hydrogels with tunable gradients using simple low voltage electric field. The gradients could be easily formed with a range of different material hydrogels. Several parameters such as solution viscosity could tune the gradient, endowing our systems with adjustability. Multiple gradients such as mechanical cues, oriented structures and different bioactive cargos were introduced to the hydrogel system simultaneously due to multifunction of the blocks, suggesting the advantages of designing complex niches. The hydrogels with mechanical gradients were fabricated to demonstrate the applications of the systems in tissue engineering. The hydrogels possessed suitable mechanical gradients to tune osteogenic-chondrogenic capacity, which was similar to that happened at the native osteochondral interface *in vivo*. Overall, the electric field-driven building blocks offer an easy and versatile strategy of developing hydrogels with multiple gradients for various complex tissues and interfacial tissue engineering.

## Materials and methods

### Fabrication of BSNF blocks and amorphous silk fibroin nanofibers (ASNF)

Beta-sheet rich silk nanofiber (BSNF) solutions were prepared according to our reported protocol (Lu et al., 2016; Ding et al., 2017.09; Wang et al., 2018b). Briefly, degummed silk was dissolved in 9.3 mol/L LiBr solution at 60 °C for 4 h, and dialyzed against distilled water for 3 d. After the dialysis, the solution was centrifuged at 9,000 rpm for 20 min at 4 °C twice to achieve silk fibroin aqueous solution with concentration of about 6 wt%. The solution was concentrated to above 20 wt% at 60 °C for more than 24 h to form metastable nanoparticles. The concentrated solution was further diluted to 2 wt% with distilled water and cultured at 60 °C until hydrogel formation. After the hydrogel formation, silk fibroin was assembled into beta-sheet rich nanofibers (BSNF). The BSNF hydrogels (2 wt%) were stirred at 1,000 rpm for 2 h at room temperature (RT), endowing with flowability. The hydrogels were centrifuged at 7,000 rpm for 15 min at 4 °C to remove bubbles.

ASNF was obtained via a dissolving-dialyzing-centrifuging processes (Dong et al., 2016; Liu et al., 2019). The degummed silk fibers were dissolved in formic acid (FA, 98%) and LiBr (8 mol/L) composite solution system with volume ratio of 1:13.32 at 60 °C for 4 h and dialyzed against distilled water for 3 d at 4 °C. The dialyzed solution was centrifuged at 9,000 rpm for 20 min at 4 °C to achieve milky solutions composed of ASNF with concentration of 1 wt%. The ASNF solution could be concentrated to 2 wt% at 60 °C for further use.

### Fabrication of hydrogels with gradient cues

**Figure 7:**
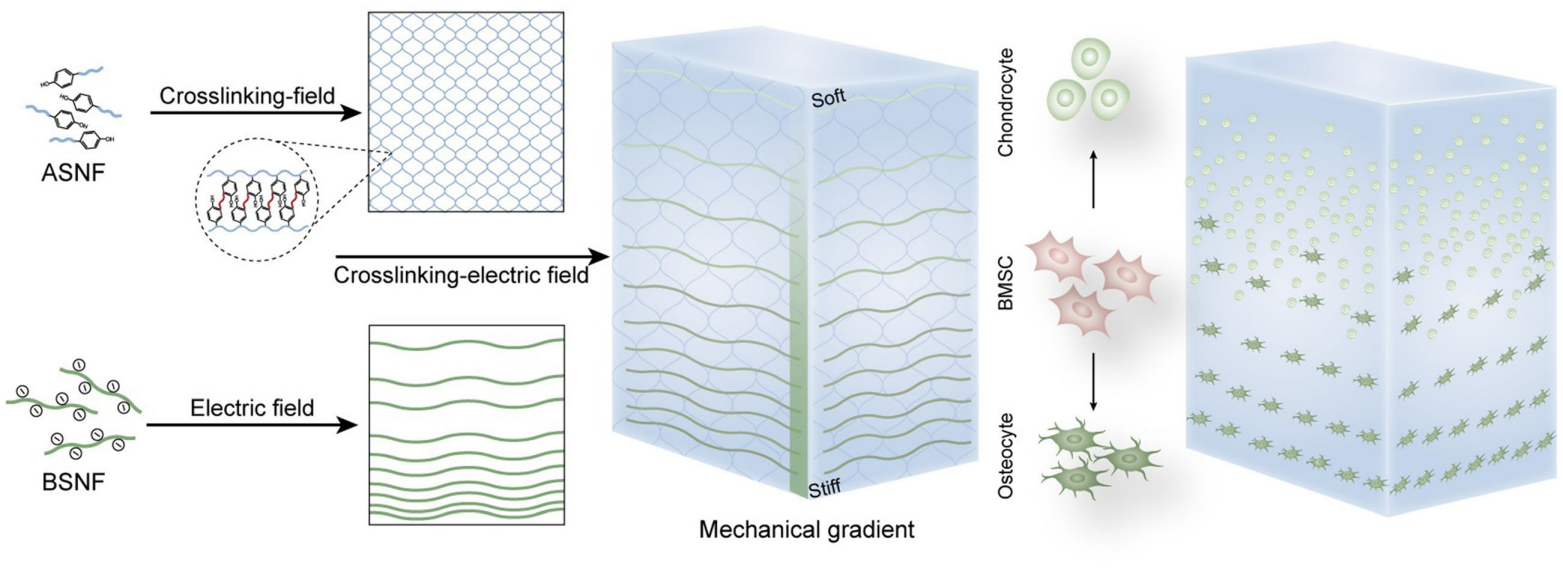
Preparation of silk nanofiber hydrogels with gradients and the control of cell differentiation

As shown in Fig. 7, ASNF (10 mL, 2 wt%) and BSNF (10 mL, 2 wt%) were blended to form composite solution. 100 μL of Horseradish Peroxidase solution (HRP, 1,000 U/mL, Sigma-Aldrich, USA) and 100 μL of H_2_O_2_ (165 mmol/L, Sigma-Aldrich, USA) were added into the composite solution (20 mL) successively and stirred for 3 min. As a typical sample, ASNF inside the mixed solution was firstly crosslinked at 37 °C for 40 min to tune the solution viscosity. Then the solution was treated under electric fields with voltage of 50 V to induce the migration of BSNF to the positive electrode (Lu et al., 2016). Meanwhile, the HRP crosslinking of ASNF continued to transform hydrogels, solidifying the gradient of BSNF. Finally, the hydrogels were cured fully at 15 min, and termed as GSNF. Along the gradient direction of BSNF, the GSNF hydrogel was divided into four parts equally. According to the content of BSNF, the divided parts were termed as GSNF1, GSNF2, GSNF3 and GSNF4, where GSNF1 had the highest amount of BSNF. As a control, pure ASNF solution (2 wt%) were also crosslinked to form hydrogels. 100 μL of HRP (1,000 U/mL, Sigma-Aldrich, USA) and 100 μL of H_2_O_2_ (165 mmol/L, Sigma-Aldrich, USA) were added into the ASNF solution (10 mL) and incubated at 37 °C for 40 min until the solid gel formation.

The BSNF gradient could be tuned through changing the crosslinking time before electrical field treatment. The above BSNF and ASNF solutions containing HRP and H_2_O_2_ were crosslinked at 37 °C for 10 min, 20 min, 40 min, 60 min and 90 min respectively, and then treated in the electrical field (50 V) to form the hydrogels with different BSNF gradients. According to the crosslinking time before electrical field treatment, the formed hydrogels were termed GSNF-E10, GSNF-E20, GSNF-E40, GSNF-E60 and GSNF-E90, respectively.

The BSNF gradients inside the hydrogels could be also regulated through tuning the ratios of ASNF and BSNF in the blend solutions. ASNF (2 wt%) and BSNF (2 wt%) were blended at different volume ratios of 3:7, 4:6, 5:5, 6:4, and 7:3, respectively. The blend solutions were crosslinked at 37 °C for 40 min to tune the solution viscosity. Then the solutions were treated under electric fields with voltage of 50 V to induce the migration of BSNF to the positive electrode. Meanwhile, the HRP crosslinking of ASNF continued to transform hydrogels, solidifying the gradient of BSNF. The formed hydrogels were termed GSNF-A3B7, GSNF-A4B6, GSNF-A5B5, GSNF-A6B4 and GSNF-A7B3 respectively.

Different bioactive cargos could be loaded on the BSNF to introduce multiple gradient cues to the hydrogels. As a model of bioactive cargos, rhodamine (50 μL, 1 mg/mL) was added to the BSNF solution and incubated overnight in dark to load on the BSNF (Lu et al., 2016). The rhodamine-loaded BSNF (BSNF, 2 wt%, 10 mL) and ASNF (10 mL, 2 wt%) were blended to form composite solution. 100 μL of (1,000 U/mL, Sigma-Aldrich, USA) and 100 μL of H_2_O_2_ (165 mmol/L, Sigma-Aldrich, USA) were added into the composite solution (20 mL) successively and stirred for 3 min. ASNF inside the mixed solution was firstly crosslinked at 37 °C for 40 min to tune the solution viscosity. Then the solution was treated under electric fields with voltage of 50 V to induce the migration of rhodamine-loaded BSNF to the positive electrode. Meanwhile, the HRP crosslinking of ASNF continued to transform hydrogels, solidifying the gradient of BSNF and rhodamine simultaneously. The formed hydrogel was termed MGH-R and equally divided into four parts that were termed MGH-R1, MGH-R2, MGH-R3 and MGH-R4, respectively.

Different crosslinking hydrogels were introduced to the system to assess universality of the strategy. Gelatin methacryloyl (Gel-MA, EFL-GM-90, Suzhou Intelligent Manufacturing Research Institute, Suzhou, China) and N-isopropylacrylamide (NIPAM, Aladdin Chemistry Co. Ltd, Shanghai, China) were chosen as the models of natural and synthetic biomaterials and blended with BSNF solution. According to previous studies (Berger et al., 2017; Gao et al., 2019), the freeze-dried Gel-MA was dissolved in lithium phenyl-2,4, 6-trimethylbenzoylphosphinate (LAP, EFL-LAP, Suzhou Intelligent Manufacturing Research Institute, Suzhou, China). The Gel-MA solution was blended with BSNF solution (2 wt%) at the volume ratio of 1:1. The blended solution was exposed to a UV light (405 nm) for 30 s to activate the crosslinking and then transferred into the electric field with the voltage of 50 V. The solution kept the UV irradiation under the electric field for 30 min, inducing the photo-crosslinking of Gel and migration of BSNF simultaneously. The hydrogels with BSNF gradients were achieved and termed MGH-M. The hydrogels were also equally divided into four parts and termed MGH-M1, MGH-M2, MGH-M3 and MGH-M4 to evaluate the gradient cues. Based on previous study (Rasib et al., 2018), N-isopropylacrylamide (NIPAM, 600 mg, Aladdin, China) and methylene-N,N-bis(acrylamide) (MBA, 60 mg, Affymetrix, China) were added to 10 mL of deionized water at room temperature. Then 10 μL of N,N,N’,N’- tetramethylethylenediamine (TEMED, Alfa Aesar, China) was added to the solution as an accelerator. The above NIPAM monomer solution (10 mL) and BSNF solution (10 mL, 2 wt%) were mixed with stirring and accelerated the crosslinking of NIPAM with 100 μL of 1% ammonium peroxodisulfate (APS). Then the mixed system was treated with the electric field (50 V) for 1 h and formed the hydrogels with the BSFN gradient. The hydrogels were termed MGH-N and also equally divided into four parts and termed MGH-N1, MGH-N2, MGH-N3 and MGH-N4 to evaluate the gradient cues.

### Characterization of Hydrogels with gradient cues

#### HRP crosslinking time

The crosslinking time was measured according to the method reported previously (Liu et al., 2019). When ASNF and BSNF were mixed with HRP (10 μl, 1,000 U/mL) and H_2_O_2_ (10 μL, 165 mmol/L) and incubated at 37 °C. The optical density (OD) at 550 nm of the system was recorded with a microplate reader (Thermo Scientific, USA) from 0 min to 90 min. The values were normalized with distilled water.

#### Viscosity of the solutions before electrical field treatment

Viscosity of the solutions before electric field treatment is a determinant of the gradient distribution. The viscosity of the solutions before the electric field treatment was measured using a digital viscometer (SNB-1, Shanghai, China) at 37 °C.

#### Fluorescence intensity of rhodamine

The rhodamine gradient was evaluated based on fluorescence intensity of rhodamine inside the hydrogels. The fluorescence intensity of rhodamine was quantified using confocal laser scanning microscope (CLSM, Olympus FV10 inverted microscope, Nagano, Japan). The date was analyzed with software of image J. Three samples were measured for each hydrogel (Lu et al., 2016).

#### Mechanical property

Mechanical properties of the hydrogels were measured with Food Texture Analyzers (TMS-Pro, FTC, USA) according to our previous studies (Han et al., 2016; Ding et al., 2016a.08; Lu et al., 2018). Hydrogels were hydrated in phosphate buffered saline (PBS) for 2 h before testing. To characterize the mechanical anisotropy of hydrogels, the samples were compressed parallel to and orthogonal to the aligned direction, respectively. The hydrogels (8 mm in diameter and 10 mm in height) were compressed by more than 30% of its original length with a 25 N load cell at the rate of 2 mm/min. Five samples were measured for each group.

#### Rheology

Rheological properties of the hydrogels were evaluated with Rheometer (AR2000, New Castle, USA)(Ding et al., 2017.09). The hydrogels were scanned in frequency sweeping mode with frequency range from 100 to 1 rad s^−1^ at 25 °C. Storage moduli (G′), loss moduli (G′′) and complex viscosity (η) were obtained using a flat plate with a 20 mm cone plate (Ti, 20/1°).

#### SEM and AFM

The microstructure of the hydrogels was characterized with Scanning Electron Microscopy (SEM, Hitachi S-4800, Hitachi, Tokyo, Japan) at 3 kV. The samples were freeze-dried and sputter-coated with gold before examination. Atom force microscopy (AFM, Nanoscope V, Veeco, NY, United States) was also used to detect the aggregation morphology of ASNF and BSNF in different parts of the GSNF hydrogels. Briefly, the GSNF hydrogels were dissolved in distilled water by shaking, diluted to 0.1% and spin coated on the surface of cleaved mica. AFM images were performed using a tapping mode at a 0.5–1 Hz scan rate according to our previous method (Dong et al., 2016; Wu et al., 2016).

#### FTIR and XRD

Fourier transform infrared spectroscopy (FTIR) and X-ray diffraction (XRD) were performed to investigate the secondary conformations of the hydrogels. For FTIR, the samples were measured with a Nicolet FTIR 5700 spectrometer (Thermo Scientific, FL, USA) in the wavenumber range from 1,750–1,450 cm^−1^. Fourier self-deconvolution (FSD) of the amide I region was used to analyze the content of various secondary conformations with peakfit software. XRD curves were measured with an X-ray diffraction (Nano ZS90, Malvern, Instruments, Malvern, U.K.). The scanning range was 5–45° with a scanning speed of 6°/min (Han et al., 2016).

### *In vitro* cell adhesion assay on hydrogels

Bone mesenchymal stem cells (BMSCs) were obtained from the femurs of male Sprague-Dawley (SD) rats (~40 g) according to our previous procedures (Ding et al., 2017.09). The use of all the SD rats was approved by the animal ethics committee of Soochow University. BMSCs were expanded in cell culture dishes in Dulbecco’s modified Eagle medium (DMEM, Gibco, Grand Island, CA, USA) supplemented with 10% FBS and 100 units/mL penicillin-streptomycin (Gibco, Grand Island, NY) at 37 °C in a 5% CO2 incubator. Cells were passaged to the third generation (P3) for further use. Hydrogel samples with diameter of 8 mm and height of 2 mm were tiled on slide glass substrates and placed in 48-well culture plates. The samples were sterilized with γ-irradiation at a dose of 25 kGy for further use.

The sterilized samples were immersed in phosphate buffer saline (PBS) solution for 2 h. Then BMSCs with 1 × 10^5^ cells/well were seeded on the surface of the samples and cultured in the medium of low glucose DMEM (Gibco, USA) for 24 h to study cell adhesion behavior *in vitro*. The samples were fixed with 4% formaldehyde (20 min), permeated with 0.2% Triton X-100 (10 min) and blocked with 2% bovine serum albumin (BSA, 30 min). Vinculin was labelled with anti-vinculin antibody (Sigma-Aldrich, St. Louis, MO) as primary antibody and Alexa-488-conjugated antibody (Invitrogen, Carlsbad, CA) as secondary antibody following the manufacturer’s protocol. F-actin was detected with tetramethylrhodamine (TRITC, Thermo Fisher, Waltham, MA), while nucleus was stained with 4,6-diamidino-2-phenyindole dilactate (DAPI, Sigma-Aldrich, St. Louis, MO). The cells were observed with a confocal laser scanning microscope (CLSM, Olympus FV10 inverted microscope, Nagano, Japan).

### Biocompatibility of the hydrogels *in vitro*

To study biocompatibility of the hydrogels *in vitro*, BMSCs with density of 1 × 10^5^ cells per well were seeded on the surface of the hydrogels. The cells were cultured for 12 days. At days 1, 3, 6, 9, and 12, the cell-seeded samples were cultured with proteinase K overnight at 56 °C to digest the hydrogels (Han et al., 2016; Liu et al., 2019). DNA content was obtained using the PicoGreen DNA assay (Invitrogen, Carlsbad, CA) according to the protocol (Han et al., 2016). The cells on the hydrogels were also observed with confocal laser scanning microscope (CLSM, Olympus FV10 inverted microscope, Nagano, Japan). When culturing for 1, 6 and 12 days, the samples were fixed with 4% formaldehyde (20 min), permeated with 0.2% Triton X-100 (10 min) and blocked with 2% bovine serum albumin (BSA, 30 min). After stained with FITC-phalloidin (Sigma-Aldrich, St. Louis, MO) and DAPI (Sigma-Aldrich, St. Louis, MO), different areas of the samples were randomly chosen and imaged with CLSM (Olympus FV10 inverted microscope, Nagano, Japan).

### Chondrogenic-osteogenic differentiation of BMSCs on GSNF hydrogels

Chondrogenic-osteogenic related gene expression and immune-fluorescence staining were used to evaluate chondrogenic and osteogenic differentiation of BMSCs on the hydrogels with stiffness gradients. P3 BMSCs (4 × 10^5^ cells/well) were seeded on the hydrogel surface in 48-well plates and cultured for 24 h with low glucose DMEM (Gibco, Thermo Fisher, MO, USA) containing 10% FBS and 100 units/mL penicillin-streptomycin (Gibco, Thermo Fisher, MO, USA). Chondrogenic-osteogenic co-culture medium supplemented with high glucose DMEM (Gibco, Thermo Fisher, MO, USA), 10% FBS, 10 ng/mL recombinant human TGF-β3 (PeproTech, Newark, NJ, USA), 100 U/mL penicillin/streptomycin (Gibco, Thermo Fisher, MO, USA), 100 nmol/L dexamethasone (Sigma-Aldrich, St. Louis, MO, USA), 91.5 μg/mL ascorbic acid 2-phosphate (Sigma-Aldrich, St. Louis, MO, USA), 10 mmol/L β-sodium glycerophosphate (Sigma-Aldrich, St. Louis, MO, USA), and 40 lg/mL L-proline (Sigma-Aldrich, St. Louis, MO, USA) was used to culture the cells for 28 days (Ribeiro et al., 2018). The co-culture medium was changed every 2 days. Quantitative real time polymerase chain reaction (PCR) was used to measure different osteogenic gene expression markers including Runt-related transcription factor 2 (Runx2), osteocalcin (OCN) and osteocalcin (OPN) (Ding et al., 2017.09; Ribeiro et al., 2018), and different chondrogenic gene expression markers such as Sry-type high mobility group box transcription factor 9 (SOX9), collagen II (COL II), and aggrecan (Acan) (Bhardwaj and Kundu, 2012). At the desired time points, total RNA from cells cultured on hydrogels was isolated using total RNA extraction kit (Tiangen Biotech, Beijing, China). 1 μg of the extracted RNA were transcribed into complementary DNA (cDNA) using a High-Capacity cDNA Reverse Transcription kit (Applied Biosystems, Carlsbad, USA). Then, relevant gene expression of each sample was measured by Quantitative real-time PCR using StepOne Plus real-time PCR system (Applied Biosystems, Foster City, USA) using a SYBR Green rapid assay kit (Applied, CA, Carlsbad, USA). The housekeeping gene glyceraldehyde-3-phosphate-dehygrogenase (GAPDH) was used as the internal control and the primer sequences were listed in Table 1.

**Table 1:** Sequences of primers used in RT-PCR

Gene	Forward primer	Reverse primer
GAPDH	F: 5’TGGGTGTGAACCACGAGAA3’	R: 5’GGCATGGACTGTGGTCATGA3’
Runx2	F: 5’CAACCACAGAACCACAAGTGC3’	R: 5’AAATGACTCGGTTGGTCTCG3’
OCN	F: 5’TATGGCACCACCGTTTAGGG3’	R: 5’GTGTGCCGTCCATACTTTCG3’
OPN	F: 5’CCAAGTAAGTCCAACGAAAG3	R: 5’GGTGATGTCCTCGTCTGTA3’
Acan	F: 5’GGCCTTCCCTCTGGATTTAG3’	R: 5’CCGCACTACTGTCCAAC3’
SOX9	F: 5’CTGAAGGGCTACGACTGGAC3’	R: 5’TACTGGTCTGCCAGCTTCCT3’
COL II	F: 5’AGGGGTACCAGGTTCTC CATC3’	R: 5’CTGCTCATCGCCGCGGTCCGA3’

After culturing on the hydrogels for 28 days, chondrogenic-osteogenic differentiation of BMSCs was identified by immunochemistry double-staining for Runx2/SOX9, OCN/COL II, and OPN/Acan, respectively. Briefly, the samples were rinsed with PBS (Hyclone, Logan, USA), fixed in a 4% paraformaldehyde solution (Sigma-Aldrich, St. Louis, MO) for 15 min and washed three times with PBS again. Then, the samples were permeabilized in 1% (*v*/*v*) Triton X-100/PBS (Sigma-Aldrich, St. Louis, MO, USA) for 5 min and further washed three times, followed by blocking treatment in 3% (*w*/*v*) BSA/PBS (BSA, bovine serum albumin, Sigma-Aldrich, St. Louis, MO, USA) for 1 h. The primary antibodies in 1% BSA/PBS were added to incubate with the samples overnight at 4 °C. After washed with PBS three times for 5 min, the samples were incubated in secondary antibody BSA/PBS solutions (1%) for 40 min at room temperature and rinsed with PBS three times for 5 min. Finally, 0.5 μg/mL of DAPI (4,6-Diamidino-2-phenylindole, Solarbio) was used to stain the nuclei for 15 min at room temperature and washed three times with PBS. Fluorescence images were collected by confocal laser scanning microscope (CLSM, Olympus FV10 inverted microscope, Nagano, Japan) and analyzed with Image J software. The list of antibodies is shown in Table S4.

### Heterotopic ossification of GSNF hydrogels *in vivo*

Twelve 8-week-old male SD rats (~300 g) were used to assess the heterotopic ossification of hydrogels. The SD rats were intraperitoneally injected with 4% chloral hydrate, and five subcutaneous pockets (left 3 and right 2) along centerline of the spine with approximately 2 cm apart were created for each rat. The GSNF-gels and ASNF-gels (8 mm in diameter and 2 mm in height) were then implanted subcutaneously into the respective pockets. At desired tie points, the implanted hydrogels along with the adjacent tissues were retrieved. The samples were fixed with 4% paraformaldehyde and embedded in paraffin. As described previously, the fixed samples were stained with hematoxylin & eosin (H&E) and also immunochemically double-stained for Runx2/SOX9, OCN/COL II, and OPN/Acan, respectively (Ding et al., 2017.09). The list of antibodies is shown in Table S1. ImageJ software was used to analyze the fluorescence intensity of specific proteins. At least three samples were performed for each group.

### Statistical analysis

All statistical analyses were performed using SPSS version 16.0 software. Comparison of mean values of the data sets was performed using one-way AVOVA and presented as mean ± standard deviations. Unless otherwise specified, *P* ≤ 0.05 was considered significant.

## Electronic supplementary material

Below is the link to the electronic supplementary material.

Supplementary material 1 (PDF 285 kb)

## References

[CR1] Aigner TB, DeSimone E, Scheibel T (2018). Biomedical applications of recombinant silk-based materials. Adv Mater.

[CR2] Berger AJ, Linsmeier KM, Kreeger PK, Masters KS (2017). Decoupling the effects of stiffness and fiber density on cellular behaviors via an interpenetrating network of gelatin-methacrylate and collagen. Biomaterials.

[CR3] Bhardwaj N, Kundu SC (2012). Chondrogenic differentiation of rat MSCs on porous scaffolds of silk fibroin/chitosan blends. Biomaterials.

[CR4] Bracaglia LG, Smith BT, Watson E, Arumugasaamy N, Mikos AG, Fisher JP (2017). 3D printing for the design and fabrication of polymer-based gradient scaffolds. Acta Biomater.

[CR5] Di Donato V, De Santis F, Albadri S, Auer TO, Duroure K, Charpentier M, Concordet JP, Gebhardt C, Del Bene F (2018). An attractive reelin gradient establishes synaptic lamination in the vertebrate visual system. Neuron.

[CR6] Ding Z, Fan Z, Huang X, Lu Q, Xu W, Kaplan DL (2016). Silk-hydroxyapatite nanoscale scaffolds with programmable growth factor delivery for bone repair. ACS Appl Mater Interfaces.

[CR8] Ding ZZ, Fan ZH, Huang XW, Bai SM, Song DW, Lu Q, Kaplan DL (2016). Bioactive natural protein-hydroxyapatite nanocarriers for optimizing osteogenic differentiation of mesenchymal stem cells. J Mater Chem B.

[CR9] Dong X, Zhao Q, Xiao L, Lu Q, Kaplan DL (2016). Amorphous silk nanofiber solutions for fabricating silk-based functional materials. Biomacromolecules.

[CR7] Ding Z, Han H, Fan Z, Lu H, Sang Y, Yao Y, Cheng Q, Lu Q, Kaplan DL (2017). Nanoscale silk-hydroxyapatite hydrogels for injectable bone biomaterials. ACS Appl Mater Interfaces.

[CR10] Engler AJ, Sen S, Sweeney HL, Discher DE (2006). Matrix elasticity directs stem cell lineage specification. Cell.

[CR11] Gao Q, Niu X, Shao L, Zhou L, Lin Z, Sun A, Fu J, Chen Z, Hu J, Liu Y (2019). 3D printing of complex GelMA-based scaffolds with nanoclay. Biofabrication.

[CR12] Han H, Ning H, Liu S, Lu QP, Fan Z, Lu H, Lu G, Kaplan DL (2016). Silk biomaterials with vascularization capacity. Adv Funct Mater.

[CR13] Hassani Besheli N, Mottaghitalab F, Eslami M, Gholami M, Kundu SC, Kaplan DL, Farokhi M (2017). Sustainable release of vancomycin from silk fibroin nanoparticles for treating severe bone infection in rat tibia osteomyelitis model. ACS Appl Mater Interfaces.

[CR14] Hubka KM, Carson DD, Harrington DA, Farach-Carson MC (2019). Perlecan domain I gradients establish stable biomimetic heparin binding growth factor gradients for cell migration in hydrogels. Acta Biomater.

[CR15] Ko E, Lee JS, Kim H, Yang SY, Yang D, Yang K, Lee J, Shin J, Yang HS, Ryu W (2018). Electrospun silk fibroin nanofibrous scaffolds with two-stage hydroxyapatite functionalization for enhancing the osteogenic differentiation of human adipose-derived mesenchymal stem cells. ACS Appl Mater Interfaces.

[CR16] Kokkinis D, Bouville F, Studart AR (2018). 3D printing of materials with tunable failure via bioinspired mechanical gradients. Adv Mater.

[CR17] Levingstone TJ, Ramesh A, Brady RT, Brama PAJ, Kearney C, Gleeson JP, O’Brien FJ (2016). Cell-free multi-layered collagen-based scaffolds demonstrate layer specific regeneration of functional osteochondral tissue in caprine joints. Biomaterials.

[CR18] Li C, Armstrong JP, Pence IJ, Kit-Anan W, Puetzer JL, Correia Carreira S, Moore AC, Stevens MM (2018). Glycosylated superparamagnetic nanoparticle gradients for osteochondral tissue engineering. Biomaterials.

[CR19] Li C, Ouyang L, Pence IJ, Moore AC, Lin Y, Winter CW, Armstrong JPK, Stevens MM (2019). Buoyancy-driven gradients for biomaterial fabrication and tissue engineering. Adv Mater.

[CR20] Liao J, Tian T, Shi S, Xie X, Ma Q, Li G, Lin Y (2017). The fabrication of biomimetic biphasic CAN-PAC hydrogel with a seamless interfacial layer applied in osteochondral defect repair. Bone Res.

[CR21] Liu J, Ding Z, Lu G, Wang J, Wang L, Lu Q (2019). Amorphous silk fibroin nanofiber hydrogels with enhanced mechanical properties. Macromol Biosci.

[CR23] Lu HH, Thomopoulos S (2013). Functional attachment of soft tissues to bone: development, healing, and tissue engineering. Annu Rev Biomed Eng.

[CR25] Lu Q, Wang X, Lu S, Li M, Kaplan DL, Zhu H (2011). Nanofibrous architecture of silk fibroin scaffolds prepared with a mild self-assembly process. Biomaterials.

[CR24] Lu Q, Bai S, Ding Z, Guo H, Shao Z, Zhu H, Kaplan DL (2016). Hydrogel assembly with hierarchical alignment by balancing electrostatic forces. Adv Mater Interfaces.

[CR22] Lu G, Ding Z, Wei Y, Lu X, Lu Q, Kaplan DL (2018). Anisotropic biomimetic silk scaffolds for improved cell migration and healing of skin wounds. ACS Appl Mater Interfaces.

[CR26] Lu X, Ding Z, Xu F, Lu Q, Kaplan DL (2019). Subtle regulation of scaffold stiffness for the optimized control of cell behavior. ACS Appl Bio Mater.

[CR27] Moller FM, Kriegel F, Kiess M, Sojo V, Braun D (2017). Steep pH gradients and directed colloid transport in a microfluidic alkaline hydrothermal pore. Angew Chem Int Ed Engl.

[CR28] Naskar D, Ghosh AK, Mandal M, Das P, Nandi SK, Kundu SC (2017). Dual growth factor loaded nonmulberry silk fibroin/carbon nanofiber composite 3D scaffolds for in vitro and in vivo bone regeneration. Biomaterials.

[CR29] Nonoyama T, Wada S, Kiyama R, Kitamura N, Mredha MT, Zhang X, Kurokawa T, Nakajima T, Takagi Y, Yasuda K (2016). Double-network hydrogels strongly bondable to bones by spontaneous osteogenesis penetration. Adv Mater.

[CR30] Oh SH, An DB, Kim TH, Lee JH (2016). Wide-range stiffness gradient PVA/HA hydrogel to investigate stem cell differentiation behavior. Acta Biomater.

[CR31] Pogoda K, Bucki R, Byfield FJ, Cruz K, Lee T, Marcinkiewicz C, Janmey PA (2017). Soft substrates containing hyaluronan mimic the effects of increased stiffness on morphology, motility, and proliferation of glioma cells. Biomacromolecules.

[CR32] Radhakrishnan J, Manigandan A, Chinnaswamy P, Subramanian A, Sethuraman S (2018). Gradient nano-engineered in situ forming composite hydrogel for osteochondral regeneration. Biomaterials.

[CR33] Rasib SZM, Ahmad Z, Khan A, Akil HM, Othman MBH, Hamid ZAA, Ullah F (2018). Synthesis and evaluation on pH- and temperature-responsive chitosan-p(MAA-co-NIPAM) hydrogels. Int J Biol Macromol.

[CR34] Ribeiro VP, da Silva Morais A, Maia FR, Canadas RF, Costa JB, Oliveira AL, Oliveira JM, Reis RL (2018). Combinatory approach for developing silk fibroin scaffolds for cartilage regeneration. Acta Biomater.

[CR35] Shen X, Zhang Y, Gu Y, Xu Y, Liu Y, Li B, Chen L (2016). Sequential and sustained release of SDF-1 and BMP-2 from silk fibroin-nanohydroxyapatite scaffold for the enhancement of bone regeneration. Biomaterials.

[CR36] Studle C, Vallmajo-Martin Q, Haumer A, Guerrero J, Centola M, Mehrkens A, Schaefer DJ, Ehrbar M, Barbero A, Martin I (2018). Spatially confined induction of endochondral ossification by functionalized hydrogels for ectopic engineering of osteochondral tissues. Biomaterials.

[CR37] Vedadghavami A, Minooei F, Mohammadi MH, Khetani S, Rezaei Kolahchi A, Mashayekhan S, Sanati-Nezhad A (2017). Manufacturing of hydrogel biomaterials with controlled mechanical properties for tissue engineering applications. Acta Biomater.

[CR38] Wang L, Lu G, Lu Q, Kaplan DL (2018). Controlling cell behavior on silk nanofiber hydrogels with tunable anisotropic structures. ACS Biomater Sci Eng.

[CR39] Wang L, Song D, Zhang X, Ding Z, Kong X, Lu Q, Kaplan DL (2018). Silk-graphene hybrid hydrogels with multiple cues to induce nerve cell behavior. ACS Biomater Sci Eng.

[CR40] Wu H, Liu S, Xiao L, Dong X, Lu Q, Kaplan DL (2016). Injectable and pH-responsive silk nanofiber hydrogels for sustained anticancer drug delivery. ACS Appl Mater Interfaces.

[CR41] Wu T, Xue J, Li H, Zhu C, Mo X, Xia Y (2018). General method for generating circular gradients of active proteins on nanofiber scaffolds sought for wound closure and related applications. ACS Appl Mater Interfaces.

[CR42] Xu F, Ma F, Ding Z, Xiao L, Zhang X, Lu Q, Lu G, Kaplan DL (2019). SERS substrate with silk nanoribbons as interlayer template. ACS Appl Mater Interfaces.

[CR43] Yang J, Liu Y, He L, Wang Q, Wang L, Yuan T, Xiao Y, Fan Y, Zhang X (2018). Icariin conjugated hyaluronic acid/collagen hydrogel for osteochondral interface restoration. Acta Biomater.

[CR44] Yin L, Wu Y, Yang Z, Denslin V, Ren X, Tee CA, Lai Z, Lim CT, Han J, Lee EH (2018). Characterization and application of size-sorted zonal chondrocytes for articular cartilage regeneration. Biomaterials.

[CR45] Zhang W, Yang G, Wang X, Jiang L, Jiang F, Li G, Zhang Z, Jiang X (2017). Magnetically controlled growth-factor-immobilized multilayer cell sheets for complex tissue regeneration. Adv Mater.

[CR46] Zhang X, Wang L, Lu Q, Kaplan DL (2018). Mass production of biocompatible graphene using silk nanofibers. ACS Appl Mater Interfaces.

