# Supplementary material for: Electric field-driven building blocks for introducing multiple gradients to hydrogels
Source: Protein Cell. 2020 Feb 12;11(4):267–85. doi: 10.1007/s13238-020-00692-z (PMC7093350; doi:10.1007/s13238-020-00692-z)
Supplement: Supplementary file 1 — Supplementary material 1 (PDF 285 kb) [file 13238_2020_692_MOESM1_ESM.pdf]

Supplemental materials

Electric Field-Driven Building Blocks for Introducing Multiple Gradients to Hydrogels

Gang Xu[a,d,#], Zhaozhao Ding[b,#], Qiang Lu[b,c,*] Xiaoyi Zhang[c], Xiaozhong Zhou[a,*], Liying Xiao[c], Guozhong Lu[b,*], David L Kaplan[e]

[a]*Department of Orthopedics, The Second Affiliated Hospital of Soochow University, Suzhou 215000, People's Republic of China*

[b]*Department of Burns and Plastic Surgery, Engineering Research Center of the Ministry of Education for Wound Repair Technology, The Affiliated Hospital of Jiangnan University, Wuxi 214041, People's Republic of China*

[c]*National Engineering Laboratory for Modern Silk & Collaborative Innovation Center of Suzhou Nano Science and Technology, Soochow University, Suzhou 215123, People's Republic of China*

[d]*Department of Orthopedics, Affiliated Hospital of Xuzhou Medical University, Lianyungang 222061,* People's Republic of China

[e]*Department of Biomedical Engineering, Tufts University, Medford, Massachusetts 02155, United States*

# The author has same contribution with the first author

Corresponding author:
Qiang Lu, Tel: (+86)-512-67061649; E-mail: Lvqiang78@suda.edu.cn
Xiaozhong Zhou, E-mail: Zhouxz@suda.edu.cn
Guozhong Lu, E-mail: Luguozhong@hotmail.com

Table S1. The mechanical properties of the GSNF and ASNF hydrogels

Direction	Mechanical properties of GSNF (kPa)				ASNF-E (kPa)
	GSNF1	GSNF2	GSNF3	GSNF4	
Perpendicular	133.4±5.2	107±6.5	64.4±10.5	22.9±2.6	17.3±2.3
Parallel	113.9±9.7	86±3.7	56.2±7.1	21.7±4.8	

Table S2. The ratio of the two compressive moduli of the GSNF hydrogels in the perpendicular and parallel electric field direction

Samples	Four prats of the GSNF hydrogels			
	GSNF1	GSNF2	GSNF3	GSNF4
GSNF-A3B7	1.13	1.08	1.06	1.02
GSNF-A4B6	1.22	1.15	1.07	1.04
GSNF-A5B5	1.17	1.14	1.08	1.06
GSNF-A6B4	1.06	1.05	1.03	1.01
GSNF-A7B3	1.03	1.02	1.02	1

Table S3. FTIR Determination of Secondary Structures of Different Hydrogels through FSD of the Amide I Region

Samples[a]	Conformation content of silk fibroin			
	β-sheet	random	α-coil	β-turn
ASNF	25.29±3.58	36.97±2.02	17.20±1.81	20.54±1.95
BSNF	55.47±1.43	22.39±3.53	12.35±4.87	9.79±5.13
GSNF-1	51.55±4.48	25.59±1.58	13.85±3.56	10.22±5.23
GSNF-2	45.28±0.54	28.41±2.12	13.08±0.61	13.23±2.05
GSNF-3	36.92±4.17	31.64±2.32	14.66±1.46	16.78±5.77
GSNF-4	29.57±8.23	34.46±7.30	16.53±0.07	19.44±4.82

[a]Five measurements per condition were obtained.

Table S4. List of immunofluorescence antibodies

Antibodies	Source	Identifier
Anti-Runx2 antibody	Abcam	ab76956
Anti-SOX9 antibody	Abcam	ab185966
Anti-OCN antibody	Abcam	ab13420
Anti-Col II antibody	Abcam	ab34712
Anti-OPN antibody	Abcam	ab8448
Anti-Acan antibody	Abcam	ab34712
goat anti-mouse IgG	Abcam	
goat anti-rabbit IgG	Abcam	
DAPI	Solarbio	C0060

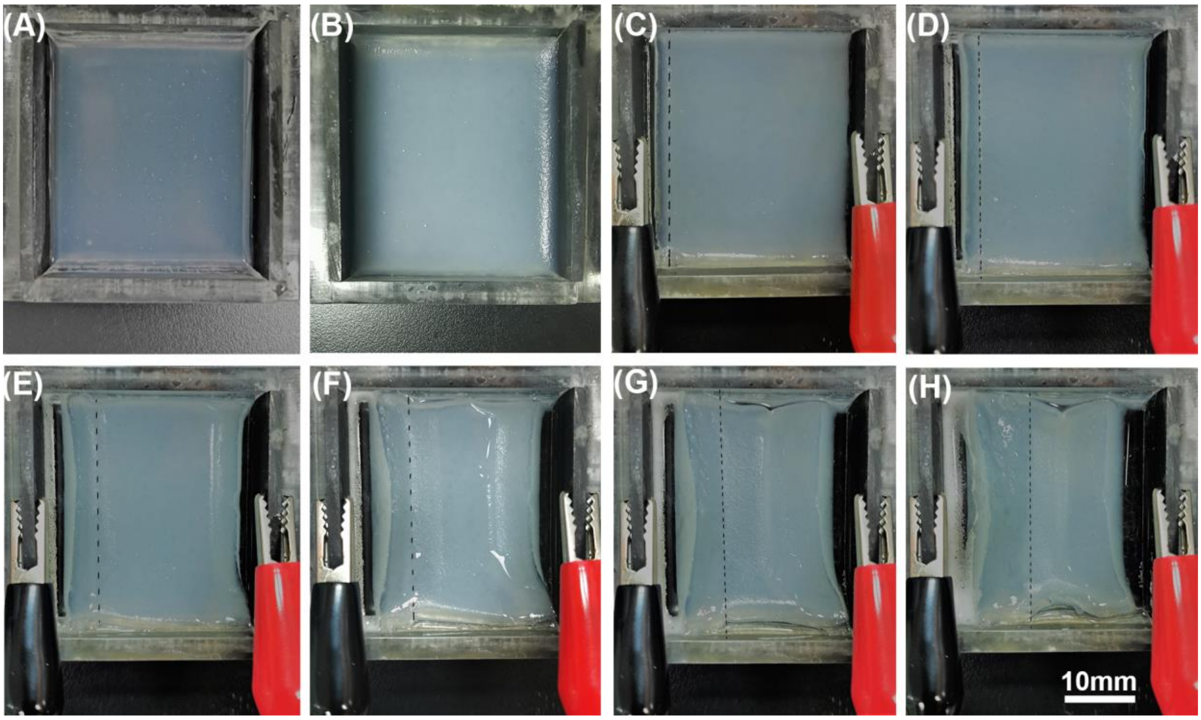

Figure S1. Visualization of hydrogel forming process in electric field: (A-B) Visualization of the HRP-crosslinked composite hydrogels (ASNF and BSNF) without electric field treatment at 0 min and 40 min; (C-H) Visualization of the HRP-crosslinked composite hydrogels (ASNF and BSNF) under electric field treatment at 0 min, 3min, 5 min, 8min, 10min and 15min. The black dotted lines indicate the migration of the BSNF in electric field alone the cathode to the anode.
